# Gait Signal Analysis with Similarity Measure

**DOI:** 10.1155/2014/136018

**Published:** 2014-07-07

**Authors:** Sanghyuk Lee, Seungsoo Shin

**Affiliations:** ^1^Department of Electrical and Electronic Engineering, Xi'an Jiaotong-Liverpool University, Suzhou 215123, China; ^2^Department of Information Security, Tongmyong University, Sinseonno, Nam-gu, Busan 608-711, Republic of Korea

## Abstract

Human gait decision was carried out with the help of similarity measure design. Gait signal was selected through hardware implementation including all in one sensor, control unit, and notebook with connector. Each gait signal was considered as high dimensional data. Therefore, high dimensional data analysis was considered via heuristic technique such as the similarity measure. Each human pattern such as walking, sitting, standing, and stepping up was obtained through experiment. By the results of the analysis, we also identified the overlapped and nonoverlapped data relation, and similarity measure analysis was also illustrated, and comparison with conventional similarity measure was also carried out. Hence, nonoverlapped data similarity analysis provided the clue to solve the similarity of high dimensional data. Considered high dimensional data analysis was designed with consideration of neighborhood information. Proposed similarity measure was applied to identify the behavior patterns of different persons, and different behaviours of the same person. Obtained analysis can be extended to organize health monitoring system for specially elderly persons.

## 1. Introduction

Analysis on the human gait signal has been studied steadily by numerous researchers [1–3]. The research on the gait signal applies to the field of healthcare development, security system, and another related area. Research methodology is based on how to classify the signal and develop pattern recognition algorithm for the comparable data sets. This algorithm applies the processing of the different signals of the same person and same action signals from multiple persons. Developing methodology for gait discrimination is challenge. However human gait signal has high dimensional characteristics, hence analyzing and designing explicit classifying formula is needed [3].

Generally, human gait signals consist of walking, sitting, standing, stepping up and down, and other usual behavior. Such a usual behavior would be done in house life, then it is quite easy for us to identify when we watch them in real situation. In order to develop a more massive monitoring system and healthcare system to analyze and identify behavior signal of each person, decision and classifying system for the gait signal is required. More specifically, decision whether he/she is doing in normal activities or not can be applied to the design of health care system. Therefore, obtained research output can be applied to the identification, healthcare, and other related fields. Additionally, more detail checking result even for the healthy people such as athletes can provide useful information whether he/she has suffered from other problems compared to the previous behavior.

To discriminate between different patterns, rational measure obtained from a statistical approach or heuristic approach is needed. By the point of statistical method, signal autocorrelation and cross correlation knowledge are useful because such formula provide us how much the signals are related with each other by the numeric value. Also, it is rather convenient to calculate due to the conventional software such as Matlab toolbox. However, it is not easy for high dimensional data to construct high dimensional correlation/covariance matrices. For heuristic approach, it needs preliminary processing for the signal, such as data redefinition and measure design based on the human thinking. Even the realization of measure based on heuristic idea is considered, ordinary measure for discrimination has to be considered such as distance. Fortunately, similarity measure design for the vague data has become a more interesting research topic; hence, numerous researchers have been focused on the similarity measure, entropy design problem for fuzzy set, and intuitionistic fuzzy set [4–8].

Similarity measure [9–12] provides useful knowledge to the clustering, and pattern recognition for data sets [13]. However, most of the conventional results were not included in high dimensional data. Human gait signal represents high dimensional data characteristics. So similarity measure design problem for high dimensional data are also needed to deal with the human gait signal. Similarity measure research has rather long history; square error clustering algorithm has been used from the late 1960s [14]. And it was modified to create the cluster program [15]. Naturally, similarity measure topic has been moved to many areas such as statistics [16], machine learning [17, 18], pattern recognition [19], and image processing. Extended research on high-dimensional data can be applied to the security business including fingerprint and iris identification, image processing enhancement, and even big data application recently.

Then, distance between vectors can be organized by norms such as 1-norm, Euclidean-norm, and so forth. Similarity measure is also designed explicitly with the distance norm. Similarity measure design problem for high-dimension needs more considerate approach. Conventionally, the similarity measure has been designed based on the distance measure between two considered data, that is, distance measure was considered information distance between two membership functions. In the similarity measure design with distance measure, measure structure should be related to the same support of the universe of discourse [14, 15]. Additionally, similarity measure consideration on overlapped or nonoverlapped data is needed because many cases of high dimensional data are represented nonoverlapped data structure. With conventional similarity measure, nonoverlapped data analysis is not possible. Hence, similarity measure design for nonoverlapped data should be followed. In order to design similarity measure on nonoverlapped data, neighbor data information was considered. Data has to be affected from the adjacent information, so similarity measure on nonoverlapped data was designed. Inside of literature, artificial data was given to compare with conventional similarity measure; calculation result was also illustrated.

In the following section, preliminary results on the similarity measure on overlapped and nonoverlapped data were introduced. Proposed similarity measure was proved and applied to overlapped and nonoverlapped artificial data. In Section 3, gait signal acquisition system was considered with sensor and data acquisition Gait signal which was also illustrated with different behaviors. High dimensional similarity measure was proposed and proved in Section 4. Similarity measure design for high dimensional data was also discussed by way of norm structure. Similarity calculation results for different behavior and individuals were also shown. Finally, conclusions are followed in Section 5.

## 2. Similarity Measure Based Distance Property

In order to design similarity measure explicitly, usual measure such as Hamming distance was commonly used as distance measure between sets *A* and *B* as follows:

(1) $$\begin{array}{c} d\left(A,B\right)=\frac{1}{n}\overset{n}{\underset{i=1}{\sum }}\left|\mu_{A}\left(x_{i}\right)-\mu_{B}\left(x_{i}\right)\right|, \end{array}$$

where *X* = {*x*_1_, *x*_2_,…, *x*_*n*_}, *μ*_*A*_(*x*_*i*_) and *μ*_*B*_(*x*_*i*_) are fuzzy membership function of fuzzy sets *A* and *B* at *x*_*i*_, and |*k*| was the absolute value of *k*. The membership function of *A* ∈ *F*(*X*) is represented by *A* = {(*x*, *μ*_*A*_(*x*))∣*x* ∈ *X*, 0 ≤ *μ*_*A*_(*x*) ≤ 1}, *X* is total set, and *F*(*X*) is the class of all fuzzy sets of *X*. Similarity measure definition was defined with the help of distance measure [14]. There are numerous similarity measures satisfying the following definition.

**Definition 1 (see [14]).**

A real function *s*: *F* ^2^ → *R* ^+^ is called a similarity measure if *s* has the following properties:

(S1) *s*(*A*, *B*) = *s*(*B*, *A*), *A*, *B* ∈ *F*(*X*);
(S2) *s*(*D*, *D* ^*C*^) = 0, *D* ∈ *P*(*X*);
(S3) *s*(*C*, *C*) = max⁡_*A*,*B*∈*F*_ *s*(*A*, *B*), *C* ∈ *F*(*X*);
(S4) *A*, *B*, *C* ∈ *F*(*X*), if *A* ⊂ *B* ⊂ *C*, then *s*(*A*, *B*) ≥ *s*(*A*, *C*) and *s*(*B*, *C*) ≥ *s*(*A*, *C*);

where *R* ^+^ = [0, *∞*), *P*(*X*) is the class of ordinary sets of *X* and *D* ^*C*^ is the complement set of *D*. By this definition, numerous similarity measures could be derived. In the following theorem, similarity measures based on distance measure is illustrated.

**Theorem 2 .**

For any set *A*, *B* ∈ *F*(*X*), if *d* satisfies Hamming distance measure, then

(2) $$\begin{array}{c} s\left(A,B\right)=d\left(\left(A\cap B\right),{\left[0\right]}_{X}\right)+d\left(\left(A\cup B\right),{\left[1\right]}_{X}\right) \end{array}$$

is the similarity measure between sets *A* and *B*.

**Proof**

Commutativity of (S1) is clear from (9) itself; that is, *s*(*A*, *B*) = *s*(*B*, *A*).

For (S2),

(3) s(D,DC)=d((D∩DC),[0]X)+d((D∪DC),[1]X)=d([0]X,[0]X)+d([1]X,[1]X)=0

is obtained because of (*D*∩*D* ^*C*^) = [0]_*X*_ and (*D* ∪ *D* ^*C*^) = [1]_*X*_, where [0]_*X*_ and [1]_*X*_ denote zero and one over the whole universe of discourse of *X*. Hence, (S2) was satisfied.

(S3) is also easy to prove as follows:

(4) s(C,C)=d((C∩C),[0]X)+d((C∪C),[1]X)=d(C,[0]X)+d(C,[1]X)=1.

It is natural that *s*(*C*, *C*) satisfied maximal value. Finally,

(5) $$\begin{array}{c} d\left(\left(A\cap B\right),{\left[0\right]}_{X}\right)=d\left(\left(A\cap C\right),{\left[0\right]}_{X}\right),\\ d\left(\left(A\cup C\right),{\left[1\right]}_{X}\right)<d\left(\left(A\cup B\right),{\left[1\right]}_{X}\right) \end{array}$$

guarantees *s*(*A*, *C*) < *s*(*A*, *B*), and

(6) $$\begin{array}{c} d\left(\left(B\cap C\right),{\left[0\right]}_{X}\right)=d\left(\left(A\cap C\right),{\left[0\right]}_{X}\right),\\ d\left(\left(A\cup C\right),{\left[1\right]}_{X}\right)<d\left(\left(B\cup C\right),{\left[1\right]}_{X}\right) \end{array}$$

also guarantees *s*(*A*, *C*) < *s*(*A*, *B*); therefore, triangular equality is obvious by the definition, and hence (S4) is also satisfied.

Besides Theorem 2, numerous similarity measures are possible. Another similarity measure is illustrated in Theorem 3, and its proof is also found in the previous result [15, 16].

**Theorem 3 .**

For any set *A*, *B* ∈ *F*(*X*), if *d* satisfies Hamming distance measure, then

(7) s(A,B)=1−d((A∩B),(A∪B)),

(8) s(A,B)=1−d(A,A∩B)−d(B,A∩B),

(9) s(A,B)=2−d((A∩B),[1]X)−d((A∪B),[0]X)

are the similarity measure between sets *A* and *B*.

**Proof**

Proofs are easy to be derived, and it was found in previous results [15, 16].

**Figure 1:**
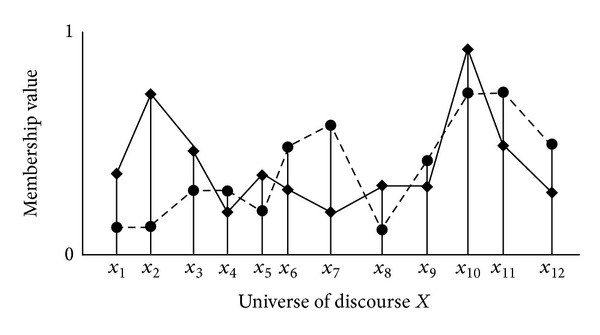
Overlapped data distribution.

**Figure 2:**
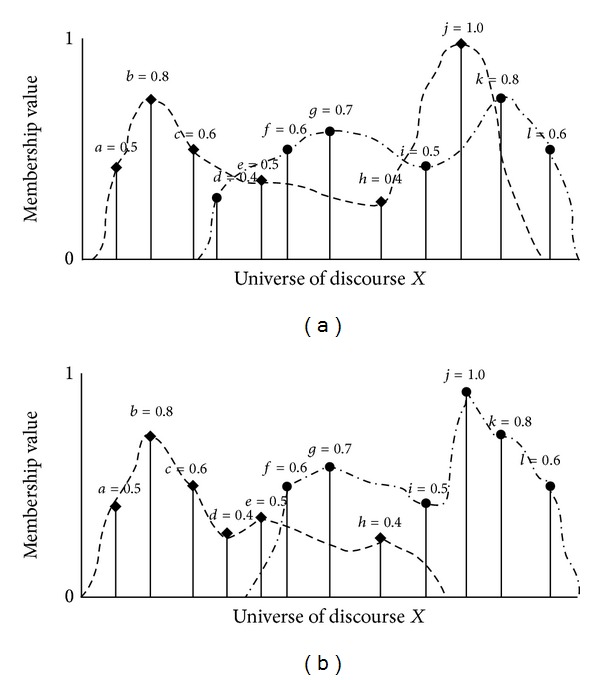
(a) Data distribution between circle and diamond. (b) Data distribution between circle and diamond.

Besides similarity measures of (7) to (9), other similarity measures are also illustrated in previous results [15–17]. With similarity measure in Theorems 2 and 3, it is only possible to compute the similarity measure for overlapped data (Figure 1). Following data distributions of diamonds (◆) and circles (●) illustrates nonoverlapped data; it is appropriate to design similarity measure for data in Figure 2. Consider the nonoverlapped data distribution of diamonds (◆) and circles (●), the similarity measure of (7) to (9) cannot provide the appropriate solution for the nonoverlapped distribution. Two data pairs that constitute different distributions are considered in Figure 2. Twelve data with six diamonds (◆) and six circles (●) are illustrated with different combination in Figures 2(a) and 2(b). Similarity degree between circles and diamonds must be different between Figures 2(a) and 2(b) because of different distribution. For example, (7) represents

(10) $$\begin{array}{c} s\left(A,B\right)=1-d\left(\left(A\cap B\right),\left(A\cup B\right)\right). \end{array}$$

From (7), *d*((*A*∩*B*), (*A* ∪ *B*)) provides distance between (*A*∩*B*) and (*A* ∪ *B*). By the following definitions:

(11) $$\begin{array}{c} A\cap B=\min\left(A,B\right),\;\;A\cup B=\max\left(A,B\right). \end{array}$$

Nonoverlapped data satisfies *A*∩*B* = min⁡⁡(*A*, *B*) = 0, and *A* ∪ *B* is defined as *A* or *B*. Hence, *s*(*A*, *B*) = 1 − (1/*N*)∑max⁡⁡(*A*, *B*) shows similarity measure, where *N* is the total number of data sets *A* and *B*. From this property,

(12) s(A,B)=1−1N∑max⁡⁡(A,B)=1−112∑(0.5+0.8+0.6+0.4+0.5+0.6+0.7        +0.4+0.5+1+0.8+0.6)=0.38,

and the same result is obtained for Figures 2(a) and 2(b). Hence, similarity measures (2) to (9) are not proper for the nonoverlapped data distribution. Two different data in Figure 2(a) are less discriminate than Figure 2(b). It means that similarity measure of Figure 2(a) has a higher value than Figure 2(b). Similar results are also obtained by the calculation of similarity measures (8) and (9).

Hence, it is required to design similarity measure for nonoverlapping data distribution. Consider the following similarity measure for nonoverlapped data such as Figures 2(a) and 2(b).

**Theorem 4 .**

For singletons or discrete data *a*, *b* ∈ *P*(*X*), if *d*satisfied Hamming distance measure, then

(13) $$\begin{array}{c} s\left(a,b\right)=1-\left|s_{a}-s_{b}\right| \end{array}$$

is a similarity measure between singletons *a* and *b*. In (13), *s*_*a*_ and *s*_*b*_ satisfy *d*((*a* ∩ **R**), [1]_*X*_) and *d*((*b* ∩ **R**), [1]_*X*_), respectively. Where **R** is whole data distribution including *a* and *b*.

**Proof**

(S1) and (S2) are clear. (S3) is also clear from definition as follows:

(14) s(C,C)=1−|sC−sC|=1−|d((C∩R),[1]X)−d((C∩R),[1]X)|=1.

Finally, (S4) *A*, *B*, *C* ∈ *F*(*X*), if *A* < *B* < *C*, then

(15) s(A,B)=1−|d((A∩R),[1]X)−d((B∩R),[1]X)|≥1−|d((A∩R),[1]X)−d((C∩R),[1]X)|=s(A,C),

because *d*((*B*∩**R**), [1]_*X*_) > *d*((*C*∩**R**), [1]_*X*_) is satisfied. Similarly, *s*(*B*, *C*) ≥ *s*(*A*, *C*) is also satisfied.

Similarity measure (13) is also designed with the distance measure such as Hamming distance. As noted before, conventional measures were not proper for nonoverlapping continuous data distributions; this property is verified by the similarity measure calculation of Figures 2(a) and 2(b).

Next, calculate the similarity measure between circle and diamond with (13).

For Figure 2(a),

(16) s(◆,●)=1−|d((◆∩R),[1]X)−d((●∩R),[1]X)|=1−1×(6|((1−0.5)+(1−0.8)+(1−0.6)      +(1−0.5)+(1−0.4)+(1−1))     −((1−0.4)+(1−0.6)+(1−0.7)      +(1−0.5)+(1−0.8)+(1−0.6))|)−1=1−16|2.3−2.4|=0.983

is satisfied.

For the calculation of Figure 2(b),

(17) s(◆,●)=1−|d((◆∩R),[1]X)−d((●∩R),[1]X)|=1−1×(6|((1−0.5)+(1−0.8)+(1−0.6)        +(1−0.4)+(1−0.5)+(1−0.4))     −((1−0.6)+(1−0.7)+(1−0.5)      +(1−1)+(1−0.8)+(1−0.6))|)−1=1−16|2.8−1.8|=0.833.

Calculation result shows that the proposed similarity measure is possible to evaluate the degree of similarity for nonoverlapped distributions. By comparison with Figure 2, distribution between diamond and circle in Figure 2(a) shows more similar.

## 3. Human Behavior Signal Analysis and Experiments

**Figure 3:**
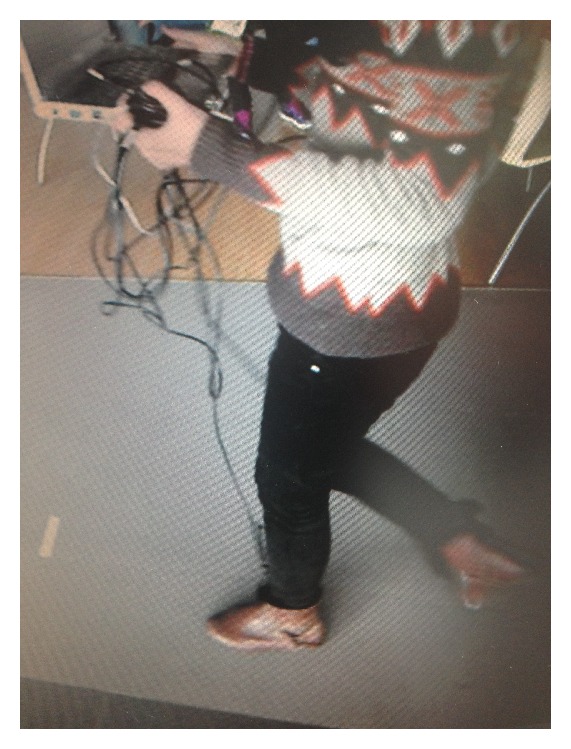
Data acquisition experiment.

Gait signals are collected with experiment unit; acquisition system (Figure 3) system is composed with all in one sensor in which accelerator, magnetic, and Gyro sensor, mobile station, and connector are integrated. Signal acquisition experiment was done as shown in the following figures.

**Figure 4:**
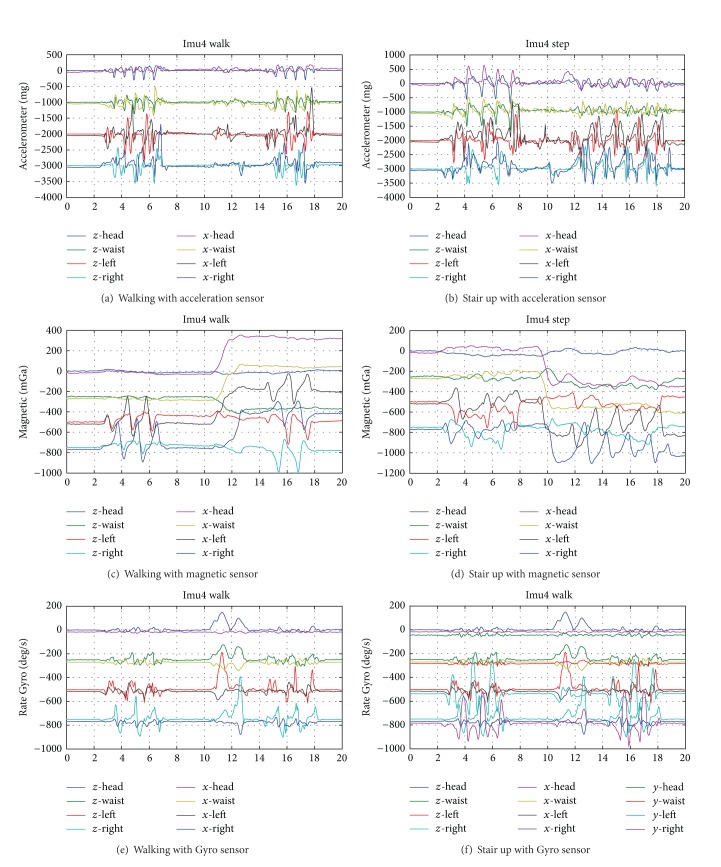
Gait signal with all in one sensor.

Gait patterns are composed of walking, step up and step down for 20 persons. For each behavior, signals are measured with all in one sensor which integrated with three sensors (accelerator, magnetic, and Gyro sensors); each sensor represents three dimension direct signals. Four sensors are attached to waist, two legs, and head. Example of obtained gait signals is illustrated in the following Figure 4. Among numerous cases, walking and stair up signals are illustrated with acceleration sensor in Figures 4(a) and 4(b), magnetic sensor in Figures 4(c) and 4(d), and Gyro sensor in Figures 4(e) and 4(f), respectively. Full signal was illustrated in Figure 4(f) for stair up with Gyro sensor; we can notice 12 signals for *x*-*y*-*z* direction, and it shows almost the same pattern for similar gait. Hence, *x*-*z* direction signals are considered in each figure. Due to the fact that signal patterns are almost the same and numerous quantity, we collect two directional signals. From the top, the first two signals represent *z*-*x* signals at head, and next ones are waist and left and right leg signals, respectively. We also carried out preprocessing to make synchronize signals and obtained gait signals that are illustrated in Figure 4.

We get the signals from the control unit, and the signal is processed in a note book. Signal characteristics were considered peak value and magnitude distance between each gait signal. Next, by the application of the similarity measure, we get the calculation of each action such as walking, step up, and so on.

## 4. Numerical Decision Calculation

### 4.1. High Dimensional Analysis

Research on big data analysis has been emphasized by research outcomes recently [7, 8, 13, 19]. Big data examples are illustrated as follows.

- Biomedical data such as DNA sequence or Electroencephalography (EEG) data. It contains not only high dimension but also large number of channel data.
- Recommendation systems and target marketing are important applications in the e-commerce area. Sets of customers/clients' information analysis help to predict their action to purchase based on customers' interest. It also includes a huge amount of data and high dimensional structure.
- Industry application such as EV station scheduling problem needs geometrical information, city size, population, traffic flow, and others. Hence, number of station and station size constitute huge data and high dimension.

Data might be expressed as high dimensional structure such as

(18)    Di=(di1,di2,di3,…,dij),where  i=1,…,n,  j=1,…,m,

where *n* and *m* denote the number of data and dimension, respectively.

Direct data comparison is applicable to overlapped data with norm definition including Euclidean norm such as

(19) d1(Di,Dj)=∑k=1m|dik−djk|, L1norm⁡,d2(Di,Dj)=∑k=1m(dik−djk)2, Euclidean-norm,dp(Di,Dj)=(∑k=1m|dik−djk|p)1/p, Lpnorm⁡.

Information distributions show various configurations, and hence it needs consider various types of distance measure to complete discriminative measure. Furthermore analysis of similarity and relation between different information should be considered carefully when it represents high-dimensional data. Specifically, *m* dimension represents the independent number of characteristics or attributes.

### 4.2. Illustrative Example

Hence, analysis and comparison with each attribute provide explicit importance of each data. Similarity measure provides analysis between patterns, such as

(20) $$\begin{array}{c} s\left(D_{i},D_{j}\right),\;\;\forall i,j=1,\ldots ,n. \end{array}$$

And comparison with different patterns for the same person is carrying out between walking, step up, or step down. Gait signal related with his/her movement was gathered to analyze their different patterns and different persons. Each signal constitutes walking, stair up, and stair down with 20 persons' gaits were gathered. Hence, personal information can be represented by multidimensional information such as

(21) $$\begin{array}{c} P_{i}=\left(W_{i},{SU}_{i},{SD}_{i}\right),\;\;\text{where}\;i=1,\ldots ,20. \end{array}$$

And among 20 personal data, 3 different behaviors were also expressed. Considering the data, it is obvious that data is overlapped. Hence, it is clear that similarity measures of (2) and (7)–(9) provide similarity calculation for overlapped data.

Similarity measure between person to person is expressed as

(22) $$\begin{array}{c} s\left(P_{i},P_{j}\right)=1-d\left(\left(P_{i}\cap P_{j}\right),\left(P_{i}\cup P_{j}\right)\right). \end{array}$$

Also different action from the same person as follows:

(23) $$\begin{array}{c} s\left(W_{i},{SU}_{i}\right)=1-d\left(\left(W_{i}\cap {SU}_{i}\right),\left(W_{i}\cup {SU}_{i}\right)\right).\; \end{array}$$

Normalized similarity calculation results are illustrated in Table 1.

Results illustrate that the stair up and down shows higher similarity than the others. However, even similarity calculation result is higher than others; it is not much close to one, it just satisfies 0.55. Due to different directions stair up/down should have basic limit to close maximum similarity.

Table 2 shows the average similarity between different individuals. Results show that the stair up is the closest even with a different gait. Naturally, walking pattern represents the least similar. In Table 3, walking similarity between different individuals is illustrated; symmetric results of 20 persons are listed in Table 3.

Similar results for stair up and down are obtained.

## 5. Conclusions

Gait signal identification was carried out through similarity measure design. Gait signal was obtained via data acquisition system including mobile station, all in one sensor attached to the head, waist, and two legs. In order to discriminate the gait signal with respect to different behaviors and individuals, similarity measure design was considered. Similarity measure was considered with the distance measure. For data distribution, overlapped and nonoverlapped distribution were considered, and similarity measure was applied to calculate the similarity. However, the conventional similarity measure was shown that it was not available to calculate the similarity on non-overlapped data. To overcome such a drawback, the similarity measure was considered with data information of neighbor. Closeness between neighbor data provides a measure of similarity among data sets; hence, the similarity measure was calculated. Calculation proposed two different artificial data, and the proposed similarity measure was useful to identify nonoverlapped data distribution. It is meaningful that similarity measure design can be extended to high dimensional data processing because gait signal was considered as a high dimensional data. With data acquisition system, 20 person gait signals were collected through experiments. Different gaits, walking, stair up, and stair down signals were obtained, and similarity measure was applied. By calculation, similarity between stair up and stair down showed higher similarity than others. Individual similarity for a different gait signal was also obtained.

Gait signal analysis can be used for behavior decision system development; it is also naturally extended to health care system, especially to elderly people. Additionally, it is also useful for athlete to provide useful information if he/she is suffering from different actions compared to previous behavior.

## Figures and Tables

**Table 1:** Similarity measure comparison between patterns.

Similarity measure	Values
*s*(*W*_*i*_, *SU*_*i*_)	0.344
*s*(*W*_*i*_, *SD*_*i*_)	0.278
*s*(*SU*_*i*_, *SD*_*i*_)	0.550

**Table 2:** Average similarity measure between individuals.

Similarity measure	Values
*s*(*PW*_*i*_, *PW*_*j*_)	0.624
*s*(*P* *SD*_*i*_, *P* *SD*_*j*_)	0.643
*s*(*P* *SU*_*i*_, *P* *SU*_*j*_)	0.712

**Table 3:** Similarity measure comparison between individuals (walking).

Similarity measure	1	2	3	4	5	6	7	⋯			18	19	20
1	1	0.511	0.621	0.420	0.462	0.581	0.617				0.581	0.470	0.623
2	0.511	1	0.592	0.590	0.490	0.632	0.543				0.603	0.619	0.577
3	0.621	0.592	1	0.510	0.611	0.640	0.599				0.710	0.566	0.582
4	0.420	0.590	0.510	1	0.701	0.653	0.589		⋯		0.612	0.629	0.585
5	0.462	0.490	0.611	0.701	1	0.599	0.603				0.489	0.581	0.620
6	0.581	0.632	0.640	0.653	0.599	1	0.576				0.499	0.545	0.629
7	0.617	0.543	0.599	0.589	0.603	0.576	1				0.710	0.627	0.634
⋮											0.590	0.611	0.589
				⋮					⋮		0.429	0.570	0.630
											0.559	0.628	0.549
18	0.581	0.603	0.710	0.612	0.489	0.499	0.710	0.590	0.429	0.559	1	0.345	0.626
19	0.470	0.619	0.566	0.629	0.581	0.545	0.627	0.611	0.570	0.628	0.345	1	0.556
20	0.623	0.577	0.582	0.585	0.620	0.629	0.634	0.589	0.630	0.549	0.626	0.556	1
